# Changes in kidney function according to ischemia type during partial nephrectomy for T1a kidney cancer

**DOI:** 10.1038/s41598-022-07919-5

**Published:** 2022-03-10

**Authors:** Junghoon Lee, Young Cheol Hwang, Sangjun Yoo, Min Soo Choo, Min Chul Cho, Hwancheol Son, Hyeon Jeong

**Affiliations:** 1 Department of Urology, Seoul National University-Seoul Metropolitan Government Boramae Medical Center, Seoul National University College of Medicine, 20, Boramae-ro 5-gil, Dongjak-gu, Seoul, 07061, Korea; 2 Department of Urology, Seoul National University Hospital, Seoul National University College of Medicine, 101, Daehak-ro, Jongno-gu, Seoul, 03080, Korea

**Keywords:** Urology, Surgical oncology

## Abstract

To compare the postoperative estimated-glomerular-filtration-rate (eGFR) and parenchymal changes between cold ischemia and zero/selective ischemia for a T1a mass. We analyzed 104 patients who underwent open partial nephrectomy with cold ischemia (53) or zero/selective ischemia (51) for T1a between 2008 and 2018 to determine postoperative renal function changes and associated factors. Postoperative renal function was expressed as (postoperative-eGFR − preoperative-eGFR)/preoperative-eGFR × 100%. Parenchymal enhancement and thicknesses of the ipsilateral kidney as tissue changes were measured on postoperative CT to identify the correlation with the renal function change. Patients with 10% or 25% decrease in eGFR were significantly more in the cold ischemia group (p = 0.032, p = 0.006). On multivariable analysis, preoperative eGFR, ischemic type, and percent change of parenchymal thickness were identified to be significantly associated with postoperative 12 months renal function (B = − 0.367, p = 0.020; B = 6.788, p = 0.042; B = 0.797, p = 0.029). Change in parenchymal thickness was negatively correlated with changes in postoperative renal function (r = − 0.277, p = 0.012). Changes in eGFR were associated with a decrease in parenchymal thickness and the type of ischemic technique. Zero/selective ischemia during partial nephrectomy may have an advantage in preserving postoperative renal function compared to cold ischemia.

## Introduction

For T1a renal masses, partial nephrectomy is strongly suggested. Numerous studies have stressed the quality of ischemia to maximize remaining renal function, and since then various techniques have been implemented^1,2^. The selective vascular clamping technique, in which the surgeon clamps the branches of renal arteries to the mass during resection, has been suggested^3,4^. The induction of ice or cold water was implemented to minimize renal damage during clamping of the renal hila^5^. In addition, partial nephrectomy without any clamping, so-called “zero ischemia” can prevent ischemic damage. The zero and selective ischemia technique would prevent changes in hyper-filtration, acceleration of nephropathy, and decrease in perfusion^2,6^.

Selective vascular clamping and off-clamping preserve renal perfusion of normal parenchyma but bleeding can be encountered during resection^7,8^. Cold ischemia technique reduces bleeding and lessens the time of renorrhaphy but can cause damage to the normal parenchyma. Dong et al. found that cold ischemia preserved ~ 7% more renal function than warm ischemia^8^. Bertolo et al. proved that postoperative renal function between off-clamp and on-clamp technique was significantly different at 12 and 24-months postoperatively^9^. And also, radiological factors relating to a renal mass, such as diameter, volume, and RENAL score, were identified to be closely related to postoperative renal function^1,8,10^. However, there exist only a few studies that provide a comparative analysis between the ischemic techniques.

We aimed to compare the ischemic techniques based on postoperative renal function. The two urologists provided data on radiological findings such as parenchymal thickness and parenchymal enhancement based on preoperative and postoperative computed tomography (CT) scan, and we also examined their correlation with postoperative renal function to uncover the effects of ischemia techniques on normal parenchyma^11,12^.

## Patients and methods

### Patient cohort

Our retrospective review was conducted of 189 patients who underwent open partial nephrectomy between 2008 and 2018. The institutional review board (IRB) of the Seoul National University-Seoul Metropolitan Government Boramae Medical Center approved this study (IRB No. 10-2019-47). Because this study retrospectively analyzed anonymized data, Informed consent was waived by the IRB. All study protocols were conducted in compliance with the principles of the Declaration of Helsinki guidelines.

Among the 189 patients, patients with clinical T1 renal mass, who underwent open partial nephrectomy, were considered suitable for the study. As a result, open partial nephrectomy was performed in 53 patients with cold ischemia and 51 patients with zero or selective ischemia. The patient cohort was divided into two groups: the cold ischemia group and zero/selective ischemia group. Serum creatinine was measured in the same center and eGFR was calculated by MDRD-2 (Modification of Diet in Renal Disease) equation. Postoperative eGFRs were compared to preoperative eGFR based on percent change of eGFR: (preoperative eGFR − postoperative eGFR)/preoperative eGFR × 100%. Serum creatinine was measured on the postoperative 3, 6, and 12 months after surgery. eGFR at 12-months postoperatively was considered as long-term follow-up eGFR. Since the decrease in renal function after partial nephrectomy is reported to be about 10% globally, we identified patients with a decrease of more than 10% in both groups^13^. And one definition of CKD progression in a KDIGO CKD guideline was described with a 25% or greater drop from baseline GFR^14^. Thus, patients with greater than 25% GFR decrement as a significant functional decline were also identified in both groups. The RENAL score was evaluated at preoperative CT within 3 months before surgery.

Before tumor resection, the kidneys were mobilized to expose the tumor to face the operator. Before each vessel clamping procedure, 100 mL of 20% d-mannitol was administered intravenously. The tumor was excised including the thin normal parenchyma after securing a safety margin of several millimeters around the tumor. The deep layer with the opened collecting system and blood vessels was closed, and the superficial layer with the resected renal stump was closed. For the cold ischemia technique, renal artery clamping was prepared while soaking cold saline with ice slush around the kidney. The main hilar vessels were entirely clamped and core cooling was maintained for 8 min. And the main hilar vessel was de-clamped after tumor resection and renorrhaphy. The zero ischemia technique was performed without hilar clamping or core cooling. For the selective ischemia, the only branch to the renal mass were exclusively clamped after all renal vessels were identified. We considered patients operated by selective ischemia technique and by zero ischemia techniques as the same group since the percent change of postoperative renal function at 12 months was not statistically distinct in both the groups.

### Radiological findings

Parenchymal enhancement and parenchymal thickness were gauged on preoperative CT and postoperative CT images taken at 6 months after surgery by two different urologists^15^. The parenchymal thickness and parenchymal enhancement were measured at the hilar level of the ipsilateral kidney without overlapping the renal mass^8^. Parenchymal enhancement was calculated on the arterial phase of 3D-kidney CT. Parenchymal enhancement was normalized by defining it as the ratio of Hounsfield unit of parenchyma to that of the aorta^11,12,16^. The parenchymal thickness and parenchymal enhancement, measured by different urologists, were averaged.

### Statistical analysis

Continuous data were expressed as means and standard deviations or median with interquartile range (IQR). Univariate and multivariable linear regression analysis were used to determine significant variables, which were correlated with postoperative eGFR. The variables with p < 0.05 on univariate analysis were dealt with on multivariate analysis and backward elimination was used. The Pearson correlation coefficients of postoperative eGFR, parenchymal enhancement, and parenchymal thickness were determined with a 95% confidence interval. A two-sided p value < 0.05 was defined as statistically significant and all data were analyzed with SPSS version 22.0.

### Ethical approval

The institutional review board of the Seoul National University-Seoul Metropolitan Government Medical Center approved this study (IRB No. 10-2019-47).

## Results

**Figure 1:**
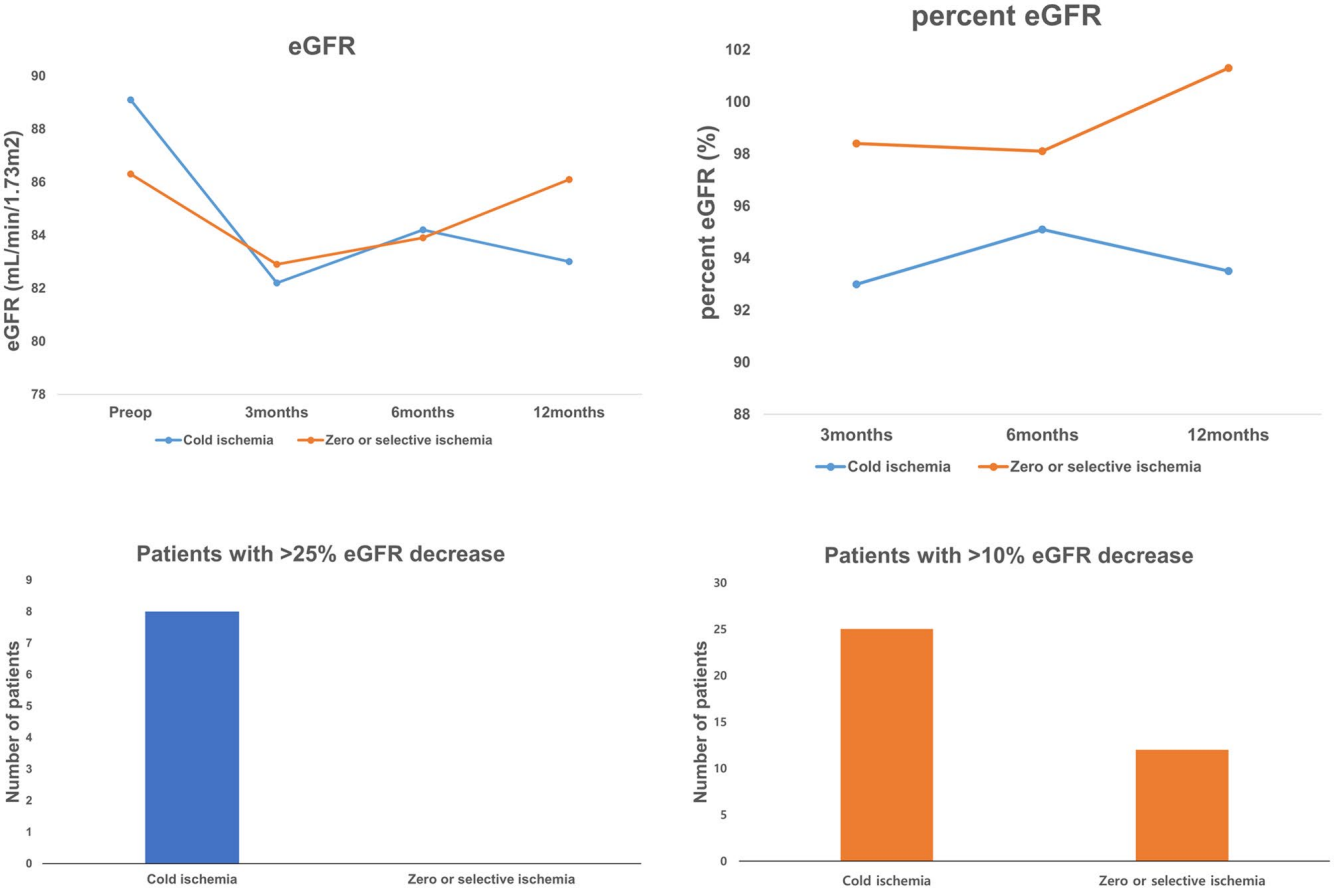
Changes of renal function according to ischemia type during partial nephrectomy. (**a**) eGFR. Preoperative and postoperative eGFR were plotted for patients grouped according to the time. (**b**) Percent change of eGFR. Percent change of eGFRs were defined as (preoperative eGFR − postoperative eGFR)/preoperative eGFR × 100% and plotted for patients grouped according to the time. Number of patients with (**c**) > 25% eGFR decrease (p < 0.006) and (**d**) > 10% eGFR decrease (p = 0.032). eGFR, estimated glomerular filtration rate.

Of the 104 patients, 53 patients (51.0%) underwent partial nephrectomy by cold ischemia technique and 51 patients (49.0%) by zero/selective ischemia. Table 1 shows the clinicopathologic characteristics of the patients. Postoperative percent change of eGFR between the two groups demonstrated statistical difference only after 12 months (cold ischemia group vs. zero/selective ischemia group: 93.0 ± 20.8 vs. 98.4 ± 20.4%, p = 0.204; 95.1 ± 21.6 vs. 98.1 ± 14.3%, p = 0.449; and 93.5 ± 20.6 vs. 101.3 ± 15.2%, p = 0.038 at 3, 6, and 12 months after the surgery, respectively) (Fig. 1). The patients with more than 10% eGFR decrease at 12 months had a significant difference with 25 and 12 patients in the cold ischemia and the zero/selective ischemia group, respectively (p = 0.032). Eight patients with more than 25% eGFR decrease were identified only in the cold ischemia group (p = 0.006). The multivariate analyses identified that preoperative eGFR, ischemic type, and percent change of parenchymal thickness were significantly related as predictive factors of percent change of postoperative eGFR at 12 months (B = − 0.367 (− 0.598 ~ − 0.137), p = 0.020; B = 6.788 (0.982–13.789), p = 0.042; B = 0.797 (0.086–1.508), p = 0.029. respectively, Table 2). Moreover, zero/selective ischemia demonstrated a more significantly preventable effect on renal damage than cold ischemia.

**Table 1:** Clinical and pathologic characteristics of patients according to ischemic type.

	Cold ischemia, median (IQR)	Zero or selective ischemia, median (IQR)	p value
Number of patients (%)	53 (50.9)	51 (49.1)	
Age (year)	60.0 (54.0–68.5)	62.0 (50.0–69.0)	0.913
**Gender, n (%)**			0.283
Male	31 (58.5)	35 (68.6)	
Female	22 (41.5)	16 (31.4)	
Hypertension, n (%)	29 (54.7)	28 (54.9)	0.985
Diabetes, n (%)	11 (20.8)	12 (23.5)	0.733
Body mass index (kg/m^2^)	24.7 (23.4–26.5)	25.1 (23.5–27.5)	0.415
Preoperative eGFR (ml/min/1.73m^2^)	92.1 (78.6–104.2)	82.4 (72.8–94.7)	0.473
Tumor volume (cm^3^)	7.8 (4.1–14.6)	3.0 (1.8–10.1)	0.49
Tumor largest diameter (cm)	2.5 (2.1–3.3)	2.1 (1.5–2.8)	0.115
Operative time (min)	110.0 (90.3–120.0)	100.0 (85.0–115.5)	0.153
Ischemia time (min)	17.0 (14.0–20.0)	0	
EBL (cc)	200.0 (150–300)	300.0 (212.5–415.0)	0.14
Transfusion, n (%)	1 (1.9)	4 (7.8)	0.156
RENAL score (range)	6 (5–7)	6 (5–7)	0.457
**Postoperative eGFR (ml/min/1.73 m**^**2**^**)**			
3 months	82.8 (67.9–98.7)	86.3 (71.5–98.0)	0.907
6 months	87.4 (67.6–98.5)	80.7 (71.0–96.8)	0.741
12 months	83.4 (69.4–96.5)	83.9 (68.6–101.0)	0.791

*eGFR* estimated glomerular filtration rate; *EBL* estimation of blood loss, *RENAL* RENAL nephrometry score.

**Table 2:** Multivariate linear regression analysis for associated factors of renal function change at postoperative 12 months after partial nephrectomy.

	Univariated analysis		Multivariable analysis	
	B (95% CI)	p value	B (95% CI)	p value
Age, year	0.158 (− 0.150 to 0.467)	0.331		
Body mass index (kg/m^2^)	− 1.132 (− 2.391 to 0.127)	0.077		
Diabetes	0.754 (− 8.890 to 10.398)	0.877		
Hypertension	6.145 (− 1.343 to 13.633)	0.097		
Preoperative eGFR (ml/min/1.73m2)	− 0.22 (− 0.400 to − 0.039)	0.018	− 0.367 (− 0.598 to − 0.137)	0.020
Ischemic time (min)	− 0.251 (− 0.687 to 0.184)	0.254		
Ischemic type	7.822 (0.435 to 15.208)	0.038	6.788 (0.982 to 13.789)	0.042
Operative time (min)	− 0.084 (− 0.227 to 0.058)	0.244		
EBL (mL)	− 0.004 (− 0.023 to 0.016)	0.717		
Tumor largest diameter (cm)	1.879 (− 2.742 to 6.500)	0.421		
Tumor volume (cm^3^)	0.179 (− 0.315 to 0.673	0.474		
RENAL score	2.796 (− 0.107 to 5.700)	0.059		
**Parenchyma thickness**				
Preoperative (mm)	− 0.756 (− 2.401 to 0.889)	0.357		
Postoperative (mm)	− 0.993 (− 2.424 to 0.439)	0.116		
Percent change (%)	− 0.822 (− 2.089 to 0.445)	0.008	0.797 (0.086 to 1.508)	0.029
**Parenchymal enhancement ratio**				
Preoperative	− 0.117 (− 0.739 to 0.505)	0.106		
Postoperative	− 0.621 (− 1.182 to − 0.060)	0.242		
Percent change (%)	0.059 (− 0.434 to 0.552)	0.595		

*B* unstandardized coefficient, *CI* confidence interval, *eGFR* estimated glomerular filtration rate, *EBL* estimation of blood loss, *RENAL *RENAL nephrometry score, *Parenchymal enhancement ratio* the ratio of the parenchyma enhancement (HU) to the aorta enhancement (HU).

We compared the radiological variables of both groups (Table 3) to explore the relationship between renal function and radiological measurements. The difference in parenchymal thickness before and after surgery and postoperative parenchymal thickness were not significantly different between the two groups (p = 0.236, p = 0.199). However, the decrement in parenchymal thickness and changes in renal function were significantly negatively correlated with postoperative renal function (R = − 0.277, p = 0.012). There was no significant difference in parenchymal enhancement between the two groups at 12 months after surgery (p = 0.579).

**Table 3:** Radiographic variables changes at postoperative 12 months after partial nephrectomy according to ischemic type. Parenchymal enhancement ratio was defined as the ratio of the parenchyma enhancement (HU) to the aorta enhancement (HU).

	Cold ischemia (n = 53), median (IQR)	Zero or selective ischemia (n = 51), median (IQR)	p value
**Parenchymal thickness**			
Preoperative (mm)	20.10 (16.10–22.50)	19.45 (16.46–21.76)	0.361
Postoperative (mm)	19.45 (15.63–21.43)	19.06 (15.68–20.86)	0.199
Difference (mm)	0.41 (0.05–1.16)	0.30 (0.01–0.71)	0.236
Percent change (%)	2.71 (0.50–5.64)	1.93 (0.07–3.40)	0.070

	Cold ischemia (n = 53), median (IQR)	Zero or selective ischemia (n = 51), median (IQR)	p value
**Parenchymal enhancement ratio**			
Preoperative	69.34 (61.61–74.89)	69.09 (36.28–74.53)	0.872
Postoperative	66.28 (60.74–71.92)	66.78 (61.71–71.76)	0.501
Difference	3.09 (0.16—7.66)	1.57 (3.67–6.33)	0.579
Percent change (%)	3.57 (0.23–10.37)	2.16 (5.55–8.49)	0.557

## Discussion

Renal ischemia types for preserving renal function in nephron sparing surgery has been a controversial topic for decades. Several factors including ischemia times of less than 25 min are known to be important for preserving renal function^4^. Many studies are being conducted on the effect of ischemic type on renal function. Several studies on robotic-assisted or laparoscopic partial nephrectomy have reported that techniques such as off-clamping, selective-clamping, or on-clamping do not affect functional outcomes^17–19^. On the contrary, some studies provide supporting evidence on the hypothesis that ischemia techniques such as cold ischemia, off-clamping, or selective vascular clamping are beneficial to preserve postoperative renal function^1,6,20–24^. Riccardo Bertolo et al. endeavored to compare the functional outcomes between off-clamped and on-clamped robot-assisted partial nephrectomy, and showed that the off-clamp is more favorable to preserve postoperative eGFR than clamping the renal hilum^9^. To preserve renal function, various ischemia techniques have been proposed, but the difference among them is not yet well understood^2,18^. Our results could support the hypothesis that zero/selective ischemia technique helps to preserve renal function even in T1a kidney cancer and could prevent a significant decrease (> 10% or > 25%) in renal function for some patients, which is comparable to the decrease in eGFR after radical nephrectomy^25^.

The main result of this study is that the zero/selective ischemia technique could be more advantageous for renal function preservation than the cold ischemia technique. The mean percent change of eGFR decrease in our study was a 6.5% decrease in cold ischemia, but a no significant decrease in zero/selective ischemia. Cold ischemia was believed to protect renal function during the longer ischemic time of up to 3 h, without causing any renal injury^26^. Funahashi et al. also showed that the cold ischemia technique allowed lengthening the ischemic time by up to 58 min without causing any renal injury^27^. However, Riccardo Bertolo et al. found that off-clamp showed a tendency towards better renal function recovery after partial nephrectomy than cold ischemia and warm ischemia technique^9^. Boga et al. also reviewed that impairment of renal function in selective renal artery clamping technique and off-clamping technique was less significant than on-clamping^22^. Our study rather argues that zero/selective ischemia technique can be regarded as superior to cold ischemia technique.

The decrement in eGFR after partial nephrectomy appeared to be affected by excising the normal functional portion of the ipsilateral kidney, ischemia-related damage, and mechanical trauma^10^. Volume loss is caused by surgical excision near the tumor margins. ischemia–reperfusion injury is caused by clamping of renal hila and traumatic damage can be caused by partial edema of renal parenchyma and alternation in vascularity near the mass. Since RENAL scores of both the patient cohorts have no statistical significance, the ischemia-related renal injury led to the difference in both the ischemia techniques, rather than the excision of normal parenchyma. The ischemia-related renal injury might lead to the difference in percent change of postoperative eGFR between both the ischemia techniques; cold ischemia reduces ischemia-related functional decline but zero/selective ischemia has almost no effect on adjacent normal tissues.

Many studies support the hypothesis that a postoperative decrease in renal function results from ischemic damage of normal parenchyma or volume reduction of normal tissue during partial nephrectomy^7,10,13,28^. Also, renal parenchymal preservation was strongly correlated with renal functional preservation^1,3,8,10,29^. However, Zhang et al. reported that the truncated parenchymal volume was more important than the atrophic change due to ischemia because the opposite pole volume did not decrease at 12 months after the on-clamping surgery^30^. Our study showed that decrement in the parenchymal thickness of normal parenchyma was negatively correlated with postoperative renal function (R = − 0.277, p = 0.012), Also, decrease in parenchymal thickness in zero/selective ischemia type was lower than in cold ischemia, although statistical significance was marginal. Since the parenchymal thickness was measured far from the tumor, we may cautiously claim that ischemic damage causes the atrophy of normal parenchyma, thereby resulting in a decline in parenchymal thickness. Zero/selective vascular clamping may help to preserve normal parenchyma and therefore provides potential benefits for functional outcomes in comparison to cold ischemia^13^. Matthias et al. showed that the off-clamping technique statistically marginally tended to maintain better microcirculation during partial nephrectomy than on-clamping^31^.

Similarly, to the outcomes of earlier studies, preoperative eGFR, RENAL score, and BMI were identified to be associated with postoperative renal function. The size was not associated with postoperative renal function in our study. The length of renal masses was so small that changes in the postoperative residual volume of the kidney probably did not significantly vary with the size^32^. Moreover, previous studies included patients with various sizes of renal tumors such as clinical T1b and T2 renal masses but our study concentrated on only clinical T1a renal tumors. Instead, the RENAL score was significantly associated with postoperative renal function^33^. Likewise, the location of renal mass had more influence on the postoperative residual volume of the kidney due to the tiny size rather than the effect of size; apparently, the postoperative renal function varied with the RENAL score. The effects of BMI on postoperative renal function were controversial, but this study supports the contention that BMI affects postoperative renal function^25^.

This study also has several limitations. First, it is a retrospective study and a small number of patients were included in this study. Therefore, preoperative factors such as RENAL score did not differ between the two groups, but potential confounding factors may not have been completely controlled. Second, we did not analyze the preserved parenchymal volume, which is often used as a quantitative predictor of renal function after partial nephrectomy using CT scan. However, it was reported that the factor of parenchymal thickness and the factor of preserved parenchymal volume were significantly related, and that the thickness was also associated with an ischemic time^8^. Third, we observed bilateral renal function by eGFR and not individual renal function by renal scintigraphy. Measurements of split renal function can compare the effects of ischemia type on the ipsilateral kidney and the contralateral kidney in a more systematic manner. This study emphasized that zero/selective ischemia type could maintains renal function more effectively than cold ischemia and drastic changes in the renal function (> 10% or > 25% decrease of eGFR) in zero/selective ischemia were significantly reduced. In the future, more number of patients can make the study extra influential and clarified. Marginal difference in parenchymal thickness, for example, might be explained more clearly.

## Conclusion

Open partial nephrectomy with selective or zero ischemia may have some benefit in preserving renal function at 12 months after surgery compared to open partial nephrectomy with cold ischemia. Atrophic changes in normal parenchyma at 12 months after surgery were significantly associated with decreased renal function, but there was no significant difference in the level of atrophic changes in normal parenchyma between the two groups. Therefore, it is necessary to analyze a larger volume of patients or to study other factors such as preserved parenchymal volume for the possibility that the postoperative difference in renal function is due to the difference in the atrophic change of the parenchyma according to the ischemic type. The choice of the zero/selective ischemia technique may have potential benefits in preserving renal function at 12 months postoperatively in patients undergoing open partial nephrectomy.

## Data Availability

The datasets generated during and/or analyzed during the current study are available from the corresponding author on reasonable request.

## References

[1] Dong W (2018). Ischemia and functional recovery from partial nephrectomy: Refined perspectives. Eur. Urol. Focus.

[2] Greco F (2019). Ischemia techniques in nephron-sparing surgery: A systematic review and meta-analysis of surgical, oncological, and functional outcomes. Eur. Urol..

[3] Volpe A (2015). Renal ischemia and function after partial nephrectomy: A collaborative review of the literature. Eur. Urol..

[4] Simone G (2015). Indications, techniques, outcomes, and limitations for minimally ischemic and off-clamp partial nephrectomy: A systematic review of the literature. Eur. Urol..

[5] Klatte T (2015). A literature review of renal surgical anatomy and surgical strategies for partial nephrectomy. Eur. Urol..

[6] Smith GL (2011). Non-clamped partial nephrectomy: Techniques and surgical outcomes. BJU Int..

[7] Sharfuddin AA, Molitoris BA (2011). Pathophysiology of ischemic acute kidney injury. Nat. Rev. Nephrol..

[8] Simmons MN (2013). Association between warm ischemia time and renal parenchymal atrophy after partial nephrectomy. J. Urol..

[9] Bertolo R (2019). Development and internal validation of a nomogram for predicting renal function after partial nephrectomy. Eur. Urol. Oncol..

[10] Simmons MN (2012). Functional recovery after partial nephrectomy: Effects of volume loss and ischemic injury. J. Urol..

[11] Kumar P (2019). Qualitative and quantitative CECT features for differentiating renal primitive neuroectodermal tumor from the renal cell carcinoma and its subtypes. Br. J. Radiol..

[12] Blomley M, Dawson P (1996). The quantification of renal function with enhanced computed tomography. Br. J. Radiol..

[13] Mir MC (2015). Decline in renal function after partial nephrectomy: Etiology and prevention. J. Urol..

[14] Levin A, Kidney disease: Improving global outcomes (KDIGO) CKD work group (2013). KDIGO 2012 clinical practice guideline for the evaluation and management of chronic kidney disease. Kidney Int. Suppl..

[15] Quinlan M (2019). Renal cell carcinoma follow-up-is it time to abandon ultrasound?. Curr. Urol..

[16] Waseda Y (2019). Predictive ability of renal cortex enhancement in dynamic computed tomography for residual renal function after nephroureterectomy: Comparison with 99mTc-diethylenetriaminopentacetic acid renography and validation study. J. Urol..

[17] Antonelli A (2022). Is off-clamp robot-assisted partial nephrectomy beneficial for renal function? Data from the CLOCK trial. BJU Int..

[18] Anderson BG (2019). Comparing off-clamp and on-clamp robot-assisted partial nephrectomy: A prospective randomized trial. Urology.

[19] Martin GL (2012). Comparison of total, selective, and nonarterial clamping techniques during laparoscopic and robot-assisted partial nephrectomy. J. Endourol..

[20] Xu J (2020). Segmental artery clamping versus main renal artery clamping in nephron-sparing surgery: Updated meta-analysis. World J. Surg. Oncol..

[21] Cacciamani GE (2019). Impact of renal hilar control on outcomes of robotic partial nephrectomy: Systematic review and cumulative meta-analysis. Eur. Urol..

[22] Boga MS, Sonmez MG (2019). Long-term renal function following zero ischemia partial nephrectomy. Res. Rep. Urol..

[23] Deng W (2018). Off-clamp partial nephrectomy has a positive impact on short- and long-term renal function: A systematic review and meta-analysis. BMC Nephrol..

[24] Mina-Riascos SH, Vitagliano G, Garcia-Perdomo HA (2020). Effectiveness and safety of partial nephrectomy-no ischemia vs warm ischemia: Systematic review and meta-analysis. Investig. Clin. Urol..

[25] Isharwal S (2018). Impact of comorbidities on functional recovery from partial nephrectomy. J. Urol..

[26] Novick AC (1983). Renal hypothermia: In vivo and ex vivo. Urol. Clin. N. Am..

[27] Funahashi Y (2014). Comparison of warm and cold ischemia on renal function after partial nephrectomy. Urology.

[28] Venkatachalam MA (2010). Acute kidney injury: A springboard for progression in chronic kidney disease. Am. J. Physiol. Renal. Physiol..

[29] Mir MC, Pavan N, Parekh DJ (2016). Current paradigm for ischemia in kidney surgery. J. Urol..

[30] Zhang Z (2015). Analysis of atrophy after clamped partial nephrectomy and potential impact of ischemia. Urology.

[31] Maruschke M (2018). Prognostic value of intraoperative measurements of renal tissue oxygenation and microcirculation on renal function in partial nephrectomy. Clin. Exp. Nephrol..

[32] Bertolo R (2019). cT1a renal masses less than 2 versus 2 cm or greater managed by robotic partial nephrectomy: A propensity score matched comparison of perioperative outcomes. J. Urol..

[33] Watts KL (2017). Value of nephrometry score constituents on perioperative outcomes and split renal function in patients undergoing minimally invasive partial nephrectomy. Urology.

